# Modulation of DNA topoisomerase II activity and expression in melanoma cells with acquired drug resistance

**DOI:** 10.1054/bjoc.1999.0947

**Published:** 2000-01-18

**Authors:** H Lage, H Helmbach, M Dietel, D Schadendorf

**Affiliations:** Institute of Pathology, Charité, Campus Mitte, Humboldt University Berlin, Schumannstr. 20/21, Berlin, D-10117, Germany; Clinical Cooperation Unit Dermatooncology at the DKFZ and Clinics Mannheim, University Heidelberg, Theodor Kutzer Ufer 1, Mannheim, D-68135, Germany

**Keywords:** melanoma, drug resistance, etoposide, cisplatin, fotemustine, vindesine

## Abstract

The role of DNA topoisomerases (Topo) IIα and IIβ was investigated in various drug-resistant melanoma cells. Melanoma cells resistant to etoposide, exhibited an up to tenfold reduced Topo II activity corresponding to an increasing degree of drug resistance indicating that modulation of Topo II activity contribute to the drug-resistant phenotype. The reduction of Topo II activity was reflected by decreased nuclear amounts of both Topo II isoforms. © 2000 Cancer Research Campaign


*British Journal of Cancer* (2000) **82**(2), 488–491
© 2000 Cancer Research Campaign
Article no. bjoc.1999.0947

### Modulation of DNA topoisomerase II activity and expression in melanoma cells with acquired drug resistance

**H Lage[1], H Helmbach[2], M Dietel[1] and D Schadendorf[2]**

[1]Institute of Pathology, Charité, Campus Mitte, Humboldt University Berlin, Schumannstr. 20/21, D-10117 Berlin, Germany; [2]Clinical Cooperation Unit Dermatooncology at the DKFZ and Clinics Mannheim, University Heidelberg, Theodor Kutzer Ufer 1, D-68135 Mannheim, Germany

**Summary** The role of DNA topoisomerases (Topo) IIα and IIβ was investigated in various drug-resistant melanoma cells. Melanoma cells resistant to etoposide, exhibited an up to tenfold reduced Topo II activity corresponding to an increasing degree of drug resistance indicating that modulation of Topo II activity contribute to the drug-resistant phenotype. The reduction of Topo II activity was reflected by decreased nuclear amounts of both Topo II isoforms. © 2000 Cancer Research Campaign

**Keywords:** melanoma; drug resistance; etoposide; cisplatin; fotemustine; vindesine

In recent years various potential mechanisms possibly involved in the development of drug resistance in melanoma cells were discussed. The exact mechanisms conferring the high intrinsic drug resistance of melanoma are currently unknown. In order to elucidate these underlying mechanisms we established a model system in vitro. Four antineoplastic drugs with different cellular targets, which are commonly used in melanoma treatment, were chosen to select stable drug-resistant sublines from the human melanoma cell line MeWo. This panel of melanoma sublines exhibiting low, various intermediate and high levels of resistance to each drug was analysed subsequently with regard to its pharmacological characteristics, cross-resistance pattern (Kern et al, 1997) and activity of DNA repair mechanisms (Lage et al, 1999).

One of the mechanisms which have been identified to contribute to resistance to antineoplastic drugs is mediated by a decreased activity of DNA topoisomerase II (Topo II) (Danks et al, 1988). This enzyme binds to double-stranded DNA, cleaves both strands, passes in an ATP-dependent manner a second strand of DNA through the cleaved site and rejoins the strands at the original site of cleavage (Osheroff et al, 1994). During breakage-reunion reaction, Topo II form a cleavable complex with DNA with the covalent linking of each Topo II subunit to each 5′-phosphoryl end of the cleaved DNA through a phosphotyrosyl bond (Liu et al, 1983). Topo II inhibitors such as etoposide interfere with the breakage-reunion reaction of Topo II by stabilizing this cleavable complex that is supposed to play the decisive role in the cytotoxic effect of Topo II-inhibiting agents (Nelson et al, 1984). The stabilized cleavable complex leads to both single- and double-strand DNA breaks, which can trigger pathways leading to cell death (Walker et al, 1991). Accordingly, resistance to Topo II-interacting agents can result from any process that leads to an altered binding of Topo II to drugs or DNA and a reduced formation of cleavable complexes.

*Received 6 May 1999*
*Revised 13 July 1999*
*Accepted 5 August 1999*

*Correspondence to:* H Lage

In mammalian cells two Topo II isoforms, the 170 kDa Topo IIα and the 180 kDa Topo IIβ exist as homodimers. Their amino acid sequences show homology at regions believed to be functionally significant (72% identical amino acid residues; Jenkins et al, 1992), suggesting a comparable mode of action. In this study, we analysed Topo IIα and IIβ expression on mRNA and protein level as well as Topo II activity in the subset of drug-resistant MeWo variants. The results demonstrate that decreasing Topo II activity closely correlates with increasing level of etoposide resistance, in contrast to measurement of Topo II expression on protein or mRNA level. Functional assays of Topo II activity may therefore be essential for a reliable prediction of the drug sensitivity.

### MATERIALS AND METHODS

### Cell lines

The human melanoma cell line MeWo (Fogh et al, 1978) and drug-resistant MeWo variants were selected in vitro and characterized as described previously (Kern et al, 1997; Lage et al, 1999). The selection conditions and the corresponding designation of the strains were as follows: MeWo untreated (MeWo); MeWo-cisplatin (MeWo CIS), 0.01 and 1.0 μg $ml^{-1}$; MeWo-vindesine (MeWo VIN), 0.5 and 5.0 ng $ml^{-1}$; MeWo-etoposide (MeWo ETO), 0.01, 0.1, 0.5 and 1.0 μg $ml^{-1}$; and MeWo-fotemustine (MeWo FOTE), 4 and 40 μg $ml^{-1}$.

### Northern blot

Northern blot analysis and polymerase chain reaction (PCR)-based generation of cDNA hybridization probes encoding human Topo IIα and IIβ were performed as described previously (Lage et al, 1999). Oligonucleotide primers used for PCR were designed after alignment with published sequences encoding Topo IIα (GenBank accession number J04088) and Topo IIβ (GenBank accession number X68060). Primers used for the amplification of Topo IIα-specific sequences were topoIIα-fw, 5′-CACAACTGGCCCTCTCTTCTGCGAC-3′ and topoIIα-rev,

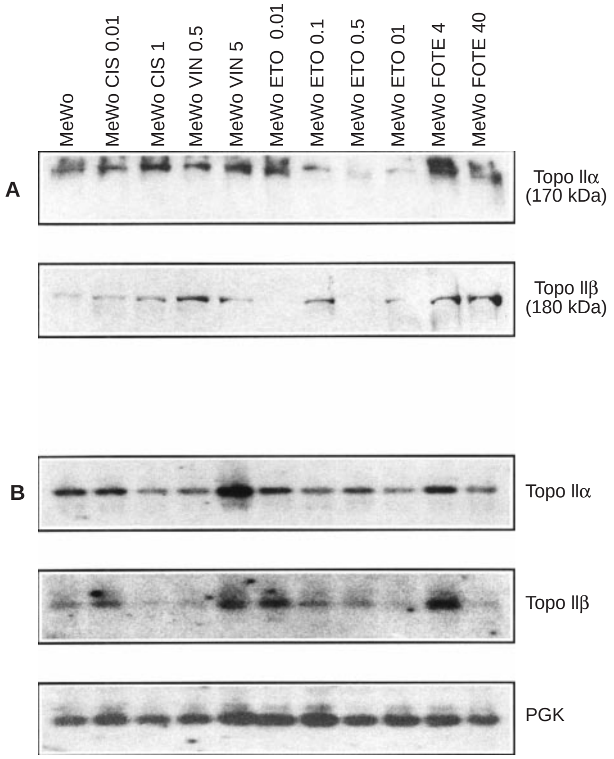

**Figure 1** Western blot analysis (**A**) of proteins prepared from crude nuclear extracts from the human melanoma cell line MeWo and the panel of drug-resistant variants harvested in the log-phase of growth. Nuclear proteins (40 μg each) were size fractionated by gel electrophoresis, transferred to a membrane, incubated with antibodies against Topo IIα and IIβ and visualized by chemiluminescence. Sizes of Topo IIα- and IIβ-specific signals were verified by comparison to molecular weight markers. Northern blot analysis (**B**) of genes encoding Topo IIα and IIβ in the human melanoma cell line MeWo and various derived drug-resistant variants. Total cellular RNA (10 μg per lane) was size fractionated on a 1% agarose gel, transferred to a nylon membrane and hybridized with $^{32}$P-labelled cDNA probes encoding Topo IIα or Topo IIβ. As control, the blots were probed with a cDNA probe encoding phosphoglycerate kinase (PGK)

5′-GGGCAACCTTTACTTCTCGCTT-3′, yielding an expected amplificate of 562 bp; and those used for amplification of Topo IIβ-specific sequences were topoIIβ-fw, 5′-ACCTGGCCAGCG-GAAAGTTTTATT-3′ and topoIIβ-rev, 5′-CCTGTTCTTTATAT-ACCTGTGTCC-3′, yielding an expected amplificate of 647 bp.

### Western blot

Western blot analysis of nuclear protein extracts was performed as described previously (Lage et al, 1999). Topo II-specific antibodies used were mouse monoclonal antibody Ki-S1 directed against Topo IIα (Boege et al, 1995) (kindly provided by Dr U Kellner, Kiel, Germany) and polyclonal rabbit antibodies directed against Topo IIβ (Boege et al, 1995) (Biotrend, Cologne, Germany).

### Topo II activity assay

Topo II catalytic activity in nuclear extracts of melanoma cells was determined using the decatenation assay (Marini et al, 1980). The standard reaction of the ATP-dependent decatenation of form I kinetoplast DNA (kDNA) from *C. fasciculata* was carried out as described previously (Kellner et al, 1997). Samples were analysed by agarose gel electrophoresis and quantified densitometrically after ethidium bromide staining.

### RESULTS

### Topo IIα mRNA and protein levels

The nuclear amount of Topo IIα protein was substantially decreased in the three etoposide-selected cell variants (MeWo ETO 0.1, MeWo ETO 0.5, MeWo ETO 1) exhibiting the highest level of drug resistance in comparison to parental MeWo cells (Figure 1A). The nuclear Topo IIα protein content was not altered in MeWo ETO 0.01 and all other lines. Levels of Topo IIα mRNA expression were analysed by Northern blot (Figure 1B). In the high level drug-resistant melanoma sublines MeWo CIS 1, MeWo ETO 1 and MeWo FOTE 40 and in MeWo ETO 0.1 a reduced Topo IIα gene expression was found.

### Topo IIβ mRNA and protein levels

No Topo IIβ protein-specific signal could be detected in the low level etoposide-resistant cell line MeWo ETO 0.01 and in MeWo ETO 0.5 (Figure 1A) indicating a considerable reduction of nuclear Topo IIβ content in these cells. Topo IIβ mRNA levels were decreased in three etoposide-resistant cell lines in parallel to the increasing level of drug-resistance exhibited (Figure 1B). Low level etoposide-resistant MeWo ETO 0.01 cells showed no significant Topo IIβ mRNA expression reduction when compared to parental cells, whereas only a weak Topo IIβ-specific signal could be detected in the high level etoposide-resistant cell line MeWo ETO 1. In addition, a decrease of Topo IIβ mRNA level was observed in the high level drug-resistant melanoma variants MeWo CIS 1 and MeWo FOTE 40 and the low level vindesine-resistant cell line MeWo VIN 0.5 compared to MeWo.

### Topo II catalytic activity

Topo II catalytic activity was assayed by decatenation of kDNA networks from *C. fasciculata* (Marini et al, 1980) using crude nuclear extracts. The degree of decatenation of kDNA by Topo II is proportional to the length of incubation. As shown in Figure 2A, Topo II activities were decreased in etoposide-resistant melanoma cells dependent on the level of etoposide resistance, as could be seen by comparing band intensities of catenated and decatenated kDNA with those of parental MeWo cells. The low level etoposide-resistant variant MeWo ETO 0.01 exhibited a Topo II decatenation activity which was similar to the activity of the parental MeWo cells, while Topo II activity was approximately fourfold lower using nuclear proteins obtained from intermediate level etoposide-resistant subline MeWo ETO 0.1. In extracts prepared from MeWo sublines exhibiting the highest level of etoposide resistance, i.e. MeWo ETO 0.5 and MeWo ETO 1, an approximately tenfold reduction of Topo II decatenation activity could be observed. Topo II catalytic activities of nuclear extracts prepared from the remaining drug-resistant melanoma cells were equivalent to parental MeWo cells (Figure 2B).

*© 2000 Cancer Research Campaign*

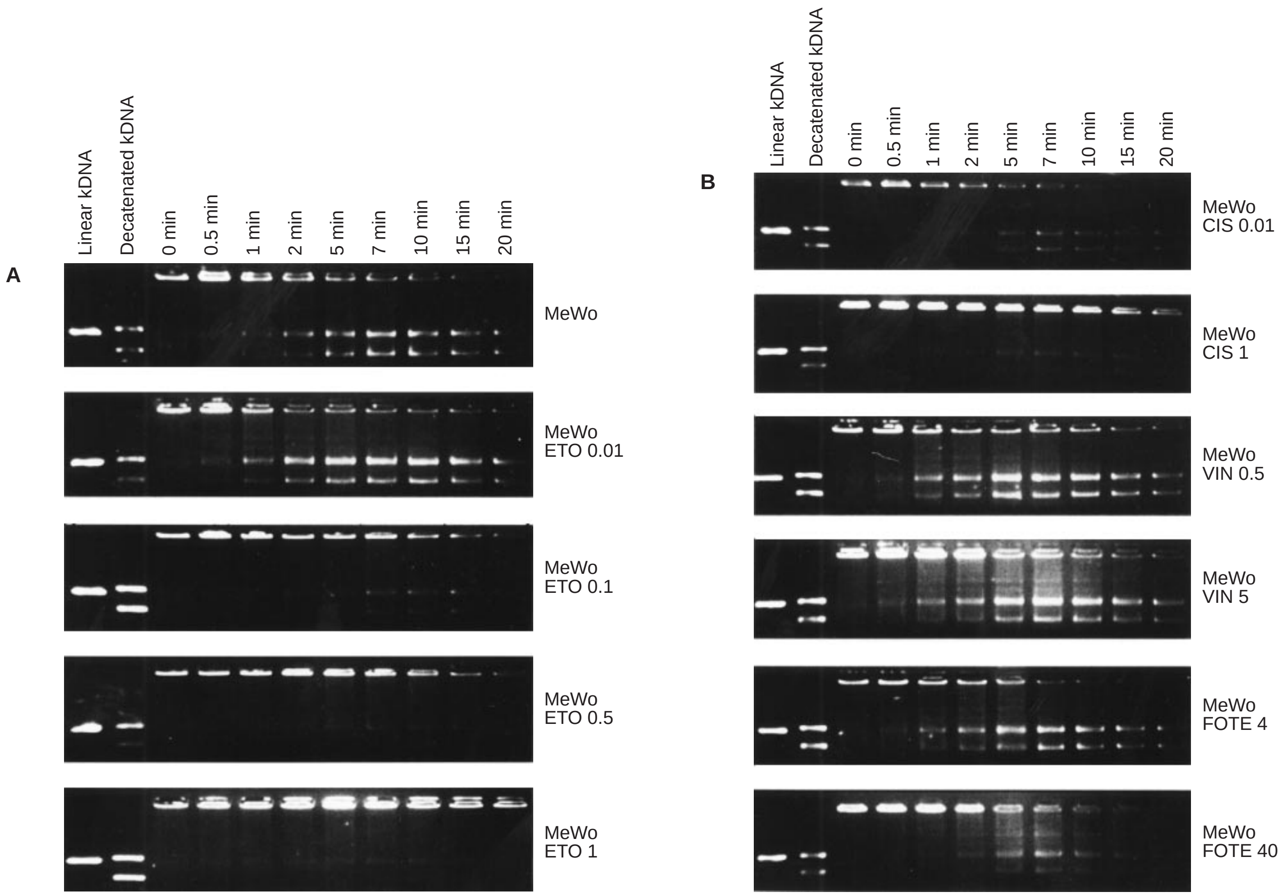

**Figure 2** Topo II activity determined in crude nuclear extracts prepared from parental melanoma cells MeWo and derived sublines selected for etoposide (**A**) and sublines resistant against cisplatin, vindesine and fotemustine (**B**). Topo II activity was measured by decatenation of kDNA networks from *C. fasciculata* as described in Materials and Methods. Reaction mixtures containing 0.4 μg nuclear proteins and 0.15 μg kDNA were incubated for various times as indicated at 37°C. The degree of decatenation of kDNA by Topo II is proportional to the length of incubation. As controls linear kDNA, digested with XhoI and complete decatenated kDNA, digested with Topo II were loaded on the gels. Near the top of the gels catenated kDNA is visible, while decatenated kDNA appears as nicked, open circular DNA (medium band) and relaxed DNA (band at the bottom of the gels)

### DISCUSSION

Melanoma is well known for its high intrinsic resistance to antineoplastic drugs used for therapy. Although various in vitro models have been established to study drug resistance of different malignancies, the mechanisms conferring resistance to melanoma cells are currently unclear. In this study we used various drug-resistant melanoma variants selected for anticancer drugs commonly applied in melanoma treatment. Melanoma cells selected for resistance against etoposide showed the broadest range of cross-resistance (Kern et al, 1997) including resistance to other Topo II inhibitors like teniposide and amsacrine (data not shown). By definition such phenotype in the absence of P-glycoprotein (data not shown), the hallmark for classical multidrug resistance (MDR), is designated as atypical MDR (Danks et al, 1988). Since Topo II represents the target molecule of etoposide, alterations of the enzyme may affect biological activity and therefore be mediating etoposide- as well as cross-resistance.

Using alternative drug-resistant in vitro systems derived from various tissues it was demonstrated that altered Topo II expression and activity led to drug resistance against Topo II inhibitors and to cross-resistance to anthracyclines and mitoxantrone. The alteration of Topo II activity can be caused by various mechanisms including reduction of the cellular amount (Liu, 1989), modulation of the catalytic activity via phosphorylation (Takano et al, 1991), or by mutations in Topo II encoding genes (Danks et al, 1993). However, it could be demonstrated that Topo II isolated from etoposide-resistant melanoma cells was much less susceptible to the drug-induced cleavable complex formation when compared to the non-resistant parental cell line (Campain et al, 1993). This phenomenon was found to be caused by a mutation in the Topo IIα-encoding gene resulting to a deletion of alanine 429. Sublines of increasing resistance were tested, and the prevalence of the mutant Topo IIα encoding mRNA increased in these cells over wild-type in parallel with their resistance to etoposide (Campain et al, 1994).

Alteration of Topo II expression has not yet reported in any drug-resistant melanoma cell line. Using a panel of drug-resistant MeWo sublines it could be demonstrated that a reduction of nuclear content of both Topo II isoforms and catalytic Topo II activity corresponded to an increasing level of drug resistance. The reduction of Topo II activity was accompanied by a decrease of the

 *© 2000 Cancer Research Campaign*

nuclear content of both Topo II isoenzymes. However, the detectable amount of nuclear Topo II proteins was not equally distributed between both Topo II isoforms. Increasing resistance level (MeWo ETO 0.1, MeWo ETO 0.5 and MeWo 1) led to a reduction of the nuclear protein content of both Topo II isoenzymes. In MeWo ETO 0.01 exhibiting the lowest level of etoposide resistance no Topo IIβ protein was detectable in contrast to unaltered Topo IIα nuclear levels. Since Topo IIα mRNA expression levels were not or only slightly altered in the etoposide-selected melanoma lines, other post-transcriptional or post-translational mechanisms might be involved in regulating Topo IIα activity, i.e. decreased protein biosynthesis or decreased transport of Topo IIα into the nucleus. Similar data were reported from a gastric carcinoma cell line showing a decreased Topo II activity (Son et al, 1998). In this cell line a reduced cellular content of Topo IIα was measured by an immunoblotting technique, but Northern blotting analysis revealed no decreased expression level of Topo IIα-encoding mRNA. Our observations have implications and clinical relevance since the data suggest that decrease of Topo II activity contributes to the development of acquired drug resistance in melanoma cells.

### ACKNOWLEDGEMENTS

This work was supported by grants of the Deutsche Forschungsgemeinschaft to D Schadendorf and H Lage (SCHA 422/7-2, LA 1039/1-2) and the Forschungsfond der Fakultät für Klinische Medizin, Mannheim (DS).

### REFERENCES

Boege F, Andersen A, Jensen S, Zeidler R and Kreipe H (1995) Proliferation-associated nuclear antigen Ki-S1 is identical with topoisomerase IIα. *Am J Pathol* **146**: 1302–1308

Campain JA, Padmanabhan R, Hwang J, Gottesman MM and Pastan I (1993) Characterization of an unusual mutant of human melanoma cells resistant to anticancer drugs that inhibit topoisomerase II. *J Cell Physiol* **155**: 414–425

Campain JA, Gottesman MM and Pastan I (1994) A novel mutant topoisomerase II alpha present in VP-16-resistant human melanoma cell lines has a deletion of alanine 429. *Biochemistry* **37**: 11327–11332

Danks MK, Schmidt CA, Cirtain MC, Suttle DP and Beck WT (1988) Altered catalytic activity of and DNA cleavage by DNA topoisomerase II from human leukemic cells selected for resistance to VM-26. *Biochemistry* **27**: 8861–8869

Danks MK, Warmoth MR, Friche E, Granzen B, Bugg BY, Harker WG, Zwelling LG, Futscher BW, Suttle DP and Beck WT (1993) Single-strand conformational polymorphism analysis of the M(r) 170,000 isoenzyme of DNA topoisomerase II in human tumor cells. *Cancer Res* **53**: 1373–1379

Fogh J, Bean MA, Brüggen J, Fogh H, Fogh JM, Hammar SP, Kodera Y, Loveless JD, Sorg C and Wright WC (1978) Comparison of a human tumor cell line before and after growth in the nude mouse. In: *The Nude Mouse in Experimental and Clinical Research*, Fogh J and Giovanella B (eds), pp. 215–234. Academic Press: New York

Jenkins JR, Ayton P, Jones T, Davies SL, Simmons DL, Harris AL, Sheer D and Hickson ID (1992) Isolation of cDNA clones encoding the β isozyme of human DNA topoisomerase II and localization of the gene to chromosome 3p24. *Nucleic Acids Res* **20**: 5587–5592

Kellner U, Hutchinson L, Seidel A, Lage H, Danks MK, Dietel M and Kaufmann SC (1997) Decreased drug accumulation in a mitoxantrone-resistant gastric carcinoma cell line in the absence of P-glycoprotein. *Int J Cancer* **71**: 817–824

Kern MA, Helmbach H, Artuc M, Karmann D, Jurgovsky K and Schadendorf D (1997) Human melanoma cell lines selected in vitro displaying various levels of drug resistance against cisplatin, fotemustine, vindesine or etoposide: modulation of proto-oncogene expression. *Anticancer Res* **17**: 4359–4370

Lage H, Christmann M, Kern MA, Dietel M, Pick M, Kaina B and Schadendorf D (1999) Expression of DNA repair proteins hMSH2, hMSH6, hMLH1, $O^6$-methylguanine-DNA methyltransferase and N-methylpurine-DNA glycosylase in melanoma cells with acquired drug resistance. *Int J Cancer* **80**: 744–750

Liu LF (1989) DNA topoisomerase poisons as antitumor drugs. *Annu Rev Biochem* **58**: 351–375

Liu LF, Rowe TC, Yang L, Tewey KM and Chen GL (1983) Cleavage of DNA by mammalian DNA topoisomerase II. *J Biol Chem* **258**: 15365–15370

Marini JC, Miller KG and Englund PT (1980) Decatenation of kinetoplast DNA by topoisomerase II. *J Biol Chem* **255**: 4976–4979

Nelson EM, Tewey KM and Liu LF (1984) Mechanism of antitumor drug action: poisoning of mammalian DNA topoisomerase II on DNA by 4′-(9-acridinyl-amino)-methanesulfon-m-aniside. *Proc Natl Acad Sci USA* **81**: 1361–1365

Osheroff N, Corbett AH and Robinson M (1994) Mechanism of action of topoisomerase II-targeted antineoplastic drugs. *Adv Pharmacol* **29**: 105–126

Son YS, Suh JM, Ahn SH, Kim JC, Yi JY, Hur KC, Hong W-S, Muller MT and Chung IK (1998) Reduced activity of topoisomerase II in an adriamycin-resistant human stomach-adenocarcinoma cell line. *Cancer Chemother Pharmacol* **41**: 353–360

Takano H, Kohno K, Ono M, Uchida Y and Kuwano M (1991) Increased phosphorylation of DNA topoisomerase II in etoposide-resistant mutants of human cancer KB cells. *Cancer Res* **51**: 3951–3957

Walker PR, Smith C, Youdale T, Leblanc J, Whitfield JF and Sikorska M (1991) Topoisomerase II-reactive chemotherapeutic drugs induce apoptosis in thymocytes. *Cancer Res* **51**: 1078–1085

*© 2000 Cancer Research Campaign* 
